# Dr. M. Elizabeth Schugens

**Published:** 1908-03

**Authors:** 


Dr. M. Elizabeth Schugens, late of Buffalo, whose death was noticed in this Journal for February, was a member of the Physicians' League, which organisation at its last meeting adopted the following memorial:

At the regular monthly meeting of the Physicians' League. of Buffalo held January 27, 1908, the following resolutions were adopted:

Whereas, God in His mercy has seen fit to take unto himself our beloved and esteemed member, M. Elizabeth Schugens, and,

Whereas, In her untimely death our League has lost a valued friend and faithful member. Therefore, be it

*Resolved*, That we ever keep before us the high ideal and example of her noble life, and be it further

*Resolved*, That these resolutions be spread upon the records of our meeting and that copies of the same be sent to the bereaved family and the Buffalo Medical Journal.

Ida C. Bender, Marie S. Wolcott,
Jane W. Carroll, Amelia Earle Trant,
Committee.

